# Biosimilars 101: considerations for U.S. oncologists in clinical practice

**DOI:** 10.1002/cam4.258

**Published:** 2014-05-09

**Authors:** Luis H Camacho, Craig P Frost, Esteban Abella, Phuong K Morrow, Sadie Whittaker

**Affiliations:** 1 St. Luke's Hospital Cancer CenterHouston, Texas; 2 St. Luke's Episcopal Hospital, Pharmacy and Oncology Service LineHouston, Texas; 3 Amgen Inc.Thousand Oaks, California

**Keywords:** Biologic, biosimilar, extrapolation of indications, interchangeability, pharmacovigilance

## Abstract

Biosimilars of biologics used for cancer treatment and supportive care are expected to enter the U.S. market soon. Biosimilars will be highly similar to their reference products, but unlike generic drugs, not identical. Differences between a biosimilar and its reference product may arise because of the complexity of biologics, and differences in the cell lines and processes used during manufacturing. Biosimilars will be approved in the United States through a regulatory pathway based on comparative analytical and clinical studies for their characterization and demonstration of no clinically meaningful differences from their reference products. Unlike generics, initial approval may not include interchangeability, as additional evidence may be required before a biosimilar could be approved as interchangeable with its reference product; interchangeable designation could allow pharmacy-level substitution without prescriber intervention. In some cases, the U.S. Food and Drug Administration (FDA) may extrapolate an indication that has not been formally investigated for the biosimilar but that is approved for the reference product. Robust safety monitoring of all biologics is important to track and accurately attribute adverse events, particularly because their inherent complexity and manufacturing differences make them susceptible to structural changes that can affect safety (e.g., immunogenicity). Accuracy of postapproval safety reports will partly depend on the biosimilar naming approach. Potential cost savings should be evaluated in the context of differences in manufacturers' patient-assistance programs, copayments, and institutional costs. A manufacturer's ability to ensure reliable supply of high-quality biosimilars should also be considered. Broad understanding of these issues is critical for oncologists preparing for their use in clinical practice.

## Introduction

Biologics are a critical component in the treatment of patients with cancer. Biosimilars—biologics that are highly similar to approved biologics—offer a potential opportunity to increase access to biologics by stimulating price competition and may lower healthcare costs 1–3. With several U.S. patents for biologics used in cancer approaching expiration, a number of companies have established biosimilar development programs for therapeutics (e.g., bevacizumab, cetuximab, rituximab, and trastuzumab) and supportive care products (e.g., epoetin alfa, filgrastim, and pegfilgrastim). When biosimilars of these medicines gain approval in the United States, some potentially very soon 1,3, they may have a significant impact on the practice of cancer medicine. Here, we provide an overview of biosimilars and discuss key considerations for practicing U.S. oncologists, including biosimilar structural complexity, manufacturing differences, regulatory approval, clinical trials, interchangeability, extrapolation of indications, safety monitoring, naming, economics, and reliability of supply. We also summarize the current biosimilar recommendations issued by the National Comprehensive Cancer Network (NCCN).

## Biosimilars Are More Complex Than Small-Molecule Drugs and Generics

**Figure 1:**
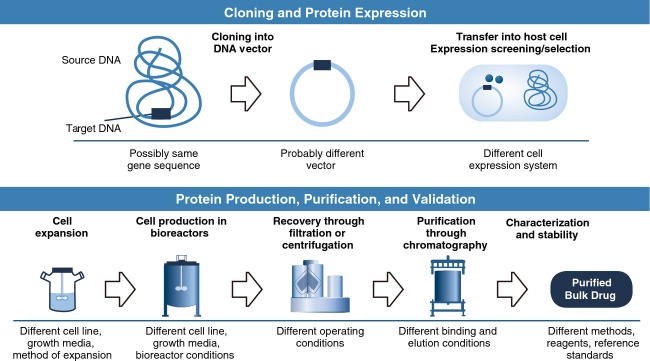
Biologic manufacturing includes multiple steps that may vary between manufacturers, potentially leading to differences between a biosimilar and its reference product that cannot be fully characterized with available analytical methods. In contrast, small-molecule drugs are manufactured through chemical reactions that can be reliably reproduced to make identical copies (generics) that can be fully characterized. Used with permission from Mellstedt H, Niederwieser D, Ludwig H. The challenge of biosimilars. *Ann Oncol*. 2008;19:412.

Biosimilars are not generic drugs because they are similar but not identical to their reference products. To understand why different manufacturers cannot produce an identical copy of a biologic, it is important to understand several key differences between biologics and small-molecule drugs. Biologics are much larger, more complex, and more sensitive to manufacturing, storage, and handling conditions, and therefore have a higher immunogenic potential compared with small-molecule drugs (Table1) 1,4,5. In addition, the manufacturing process for biologics is more complex than for small-molecule drugs, requiring multiple steps for cloning; selecting, maintaining, and expanding the cell line; and isolating, purifying, and characterizing the product (Fig.1). Small-molecule drugs are synthesized through a series of predictable chemical reactions that can be reliably reproduced to yield identical copies (i.e., generics) 1. Biologics are manufactured using cell lines and production processes developed by each manufacturer, making it challenging for different manufacturers of biosimilars to develop identical copies of biologics. Manufacturing differences between a biosimilar and its reference product can lead to differences in molecular structure (e.g., glycosylation), content (e.g., isoforms, impurities, and aggregates), biological activity, and immunogenicity. Although many of these differences can be characterized with current analytical techniques, others cannot 5. The potential for such changes to affect clinical safety and efficacy should be evaluated in clinical trials, as reflected in the current regulatory approval standards for biosimilars in both the European Union (EU) and the United States 6,7.

**Table 1:** Differences in characterization and manufacturing of biologics and small-molecule drugs.^1^

	Biologics (protein-based drugs)	Small molecules (chemically based drugs)
Properties		
Size	Large	Small
Structure	Complex	Simple
Degradation mechanism	Complex	Precise and known
Variability	Heterogeneous product	Single, defined structure
Manufacturing	Unique bank of living cells Unlikely to achieve identical copy	Predictable chemical and reagent reaction Identical copy can be made
Characterization	Difficult to fully characterize	Easy to fully characterize
Stability	More sensitive to storage and handling conditions	Less sensitive to storage and handling conditions
Immunogenicity	Higher potential	Lower potential

1 Biologics are protein-based drugs and can be thousands of times larger than chemically based small-molecule drugs. The amino acid chains of biologics form complex multidimensional structures. Additionally, biologics may have variations in protein folding, subunit makeup, and posttranslational modification (e.g., glycosylation), whereas small-molecule drugs have well-defined chemical structures. Thus, biologics have a higher immunogenic potential than small molecule drugs and are more sensitive to storage and handling conditions 1,4,5. Although small-molecule drugs can be fully characterized using current analytical procedures, it is much more difficult to fully characterize biologics because they comprise a heterogeneous mixture of related molecules 5. Manufacturing of biologics is more complex than that of small-molecule drugs, and differences in cell lines and manufacturing processes for biologics make it unlikely for different manufacturers to make identical copies of a biologic. In contrast, identical copies of small-molecule drugs can be synthesized through predictable chemical reactions.

## Assessing Safety and Efficacy of Biosimilars: Review of Regulatory Guidance in the EU and United States

Because biologics are more complex than small-molecule drugs and because biosimilars are similar but not identical to their reference products, regulatory authorities around the world have recognized the need for a unique approval pathway for biosimilars that is distinct from the approval pathway for generics and that addresses these differences 8–10. The first biosimilar approval pathway was established in the EU, and because it has generally been considered successful 11,12, it has served as a reference for other national regulatory authorities around the world, including the United States (reviewed below) 7,8,13–15.

### Biosimilar regulation in the EU

The EU was the first to establish a regulatory pathway for biosimilars because of the timing of patent expirations for several biologics (epoetin alfa, filgrastim, and somatropin). A 2001 EU directive provided the legal basis for the approval of biosimilars in the EU 16, allowing the European Medicines Agency (EMA) to draft the first biosimilar guideline, which came into effect in 2005 and outlines the basic principles of the EU biosimilars regulatory approach: comparing the proposed biosimilar with its reference product in analytical and clinical studies to demonstrate similarity with respect to quality, safety, and efficacy 9. In the context of biotherapeutics, “quality” encompasses the product and manufacturing process to ensure the drug is pure, safe, efficacious, and consistent. The EMA adopted biosimilar guidelines in 2006 that address the following: (1) quality issues in analytical studies (e.g., selection of a reference product, methods, product characterization, and demonstration of biological activity and purity) 17 and (2) nonclinical and clinical issues (e.g., pharmacotoxicological assessment, pharmacokinetics, pharmacodynamics, efficacy, and safety). These guidelines also address the need for pharmacovigilance (postapproval safety monitoring) of the biosimilar and a risk-management plan emphasizing immunogenicity 18. The three original biosimilar guidelines have been revised to reflect biosimilar experience gained in the EU since their initial adoption; at this time, potential revisions of the existing guidelines are under review 19–21. Unique to the EU regulatory approach, the EMA has also developed class-specific biosimilar guidelines, including guidelines on nonclinical and clinical studies of recombinant therapeutic proteins, recombinant erythropoietins, interferon *β*, and monoclonal antibodies 18,22,23.

The first biosimilar was authorized for use in the EU in 2006 12. Currently, nine distinct biosimilars have marketing authorization under 16 different trade names (two recombinant human erythropoietin products, four granulocyte colony-stimulating factor [G-CSF] products, one human growth hormone product, one human follicle-stimulating hormone product, and one recently approved monoclonal antibody against tumor necrosis factor α; two products were withdrawn at the request of the marketing authorization holder [Table2]) 12. The EMA approval process recognizes the importance of rigorous analytical testing of biosimilars supported by appropriate confirmatory clinical evidence to evaluate the clinical impact of minor changes in structure compared with the reference product. This is highlighted by the refusal of a candidate biosimilar (recombinant human interferon *α*-2a) 24. Although the biosimilar candidate was claimed by the sponsor to be similar to the reference biologic in analytical testing, the products displayed different impurity profiles, and the EMA assessors concluded that the data were incomplete and inconclusive. Furthermore, clinical trials of this product revealed differences in pharmacokinetics and clinical efficacy (hepatitis C virus infection relapse rate) compared to its reference product 24.

**Table 2:** Biosimilars that have received marketing authorization in the EU.^1^

Biosimilar name	Biosimilar active substance	Status (authorization or refusal date)	Manufacturer	Marketing authorization holder/applicant
*Human erythropoietin products*				
Abseamed	Epoetin alfa	Authorized (2007)	Rentschler Biotechnologie GmbH Lek Pharmaceuticals	Medice Arzneimittel Pütter GmbH & Co. KG
Binocrit	Epoetin alfa	Authorized (2007)	Rentschler Biotechnologie GmbH Lek Pharmaceuticals	Sandoz GmbH
Epoetin Alfa Hexal	Epoetin alfa	Authorized (2007)	Rentschler Biotechnologie GmbH Lek Pharmaceuticals	Hexal AG
Retacrit	Epoetin zeta	Authorized (2007)	Norbitec GmbH	Hospira UK Ltd.
Silapo	Epoetin zeta	Authorized (2007)	Norbitec GmbH	Stada Arzneimittel AG
*Human granulocyte colony-stimulating factor products*				
Biograstim	Filgrastim	Authorized (2008)	SICOR Biotech UAB	CT Arzneimittel GmbH
Filgrastim Hexal	Filgrastim	Authorized (2009)	Sandoz GmbH	Hexal AG
Filgrastim ratiopharm	Filgrastim	Withdrawn^2^ (2008)	SICOR Biotech UAB	Ratiopharm GmbH
Grastofil	Filgrastim	Authorized (2013)	Intas Biopharmaceuticals Ltd. Apotex Nederland BV	Apotex Europe BV
Nivestim	Filgrastim	Authorized (2010)	Hospira Zagreb	Hospira UK Ltd.
Ratiograstim	Filgrastim	Authorized (2008)	SICOR Biotech UAB	Ratiopharm GmbH
Tevagrastim	Filgrastim	Authorized (2008)	SICOR Biotech UAB	Teva Generics GmbH
Zarzio	Filgrastim	Authorized (2009)	Sandoz GmbH	Sandoz GmbH
*Human growth hormone products*				
Omnitrope	Somatropin	Authorized (2006)	Sandoz GmbH	Sandoz GmbH
Valtropin	Somatropin	Withdrawn^3^ (2006)	LG Life Sciences Ltd.	BioPartners GmbH
*Human interferon α-2a product*				
Alpheon	Recombinant human interferon *α*-2a	Refused (2006)	Rentschler Biotechnologie GmbH	BioPartners GmbH
*Human follicle-stimulating hormone product*				
Ovaleap	Follitropin alfa	Authorized (2013)	Merckle Biotec GmbH Teva Pharmaceuticals Europe BV	Teva Pharma BV
*Anti-human tumor necrosis factor α 2 monoclonal antibody*				
Inflectra	Infliximab	Authorized (2013)	Celltrion Inc.	Hospira UK Ltd
Remsima	Infliximab	Authorized (2013)	Celltrion Inc.	Celltrion Healthcare Hungary Kft

1 Information on clinical trials for these products is available in their respective assessment histories in the European Public Assessment Reports 12.

2 Marketing authorization in the EU withdrawn in 2011 at the request of the marketing authorization holder.

3 Marketing authorization in the EU withdrawn in 2012 at the request of the marketing authorization holder.

### Evolving regulation of biosimilars in the United States

**Figure 2:**
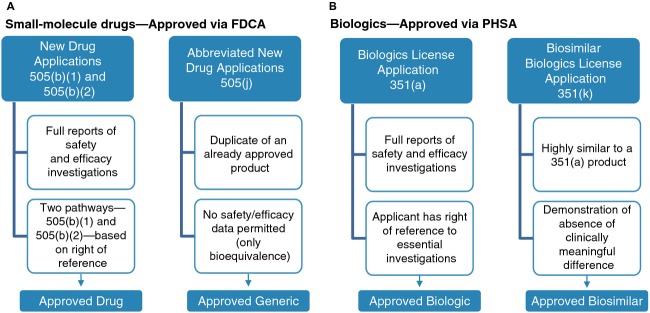
Approval pathways for (A) small-molecule drugs versus generics and (B) biologics versus biosimilars. New small-molecule drugs are approved under a New Drug Application as authorized by the Food, Drug, and Cosmetic Act (FDCA). A subsequent generic drug can be approved via an Abbreviated New Drug Application that demonstrates that it is a duplicate of the small-molecule reference drug (i.e., same active ingredient, strength, dosage form, route of administration, and conditions of use; bioequivalent). In contrast, new biologics are evaluated and approved under a 351(a) Biologics License Application as authorized by the Public Health Service Act (PHSA). The Biologic Price Competition and Innovation Act of 2009 created a 351(a) biosimilar Biologics License Application pathway that requires demonstration that the biosimilar is highly similar to its biologic reference product, notwithstanding minor differences in clinically inactive components, and that it has no clinically meaningful differences from its reference product in terms of safety, purity, and potency 10.

The Biologics Price Competition and Innovation (BPCI) Act of 2009 is part of the Patient Protection Affordable Care Act that was signed into U.S. law in 2010. The BPCI Act provides an abbreviated regulatory pathway for biosimilars via a 351(k) application (Fig.2) 2,7,10,14,15, which is designed to reduce the amount of testing required in animals and humans compared with innovator biologics that are approved through a 351(a) Biologics License Application 2. This abbreviated pathway for biosimilars is distinct from the regulatory pathway for generic small-molecule drugs, which is based on demonstrating pharmaceutical equivalence and pharmacokinetic equivalence with its small-molecule reference product 25. The only human study necessary for approval of a generic, therefore, is a pharmacokinetic equivalence study in healthy volunteers. In comparison, approval of a biosimilar requires evidence that it is highly similar to its biologic reference product, notwithstanding minor differences in clinically inactive components, and that it produces no clinically meaningful differences from its reference product in terms of safety, purity, and potency 10. Assessment of these parameters in analytical and clinical studies to demonstrate similarity is described below.

The U.S. Food and Drug Administration (FDA) has issued three draft guidance documents that outline the U.S. regulatory approach for biosimilars. This approach is based on evidence submitted by the biosimilar manufacturer 7,14,15 and includes analytical and clinical studies comparing the proposed biosimilar to an approved biologic reference product 14. Comparative analytical studies are recommended to characterize primary, secondary, tertiary, and quaternary structures; posttranslational modifications; and functional activity, as well as to identify possible product- and process-related impurities. The FDA recommends comparative assessment of toxicity in animal models, followed by comparative clinical pharmacokinetic and pharmacodynamic studies (if a relevant pharmacodynamic measure is available), and ultimately, clinical confirmation of efficacy as well as assessment of safety and immunogenicity 7. Such clinical trials are important because the clinical consequences of structural differences between a biosimilar and its reference product are unknown 26; small differences in structure (e.g., variations in glycosylation) potentially affect the safety and efficacy of a recombinant protein 7. The development of neutralizing antibodies among two patients with chronic kidney disease treated with HX575, a biosimilar version of epoetin alfa, resulted in premature termination of the trial in the EU and reinforces the importance of clinical trials evaluating the safety of biosimilars (see section Pharmacovigilance: safety monitoring after approval below) 27.

In the United States, a biosimilar may be further evaluated for interchangeability with the reference product—an option that is unique to the FDA regulatory pathway 10. Interchangeability is a higher standard of biosimilarity 14. A biosimilar designated as interchangeable is “expected to produce the same clinical result as the reference product in any given patient,” and if “administered more than once to an individual, the risk in terms of safety or diminished efficacy of alternating or switching between use of the biological product and the reference product is not greater than the risk of using the reference product without such (a) switch” 10. The FDA has not yet publicly defined the type(s) of clinical studies that will be required to demonstrate interchangeability. The BPCI Act states that an interchangeable biologic “may be substituted for the reference product without the intervention of the healthcare provider who prescribed the reference product” 10. Notably, measures to facilitate accurate medical records in the event of an automatic substitution (e.g., recordkeeping requirements, the use of interoperable electronic health records, and/or prescriber notification after the fact) depend on each state's laws, which determine this policy (see section Interchangeability and automatic substitution below).

### Biosimilar clinical trials

Biosimilar clinical trials may pose distinct challenges for sponsors and patients that should be considered. In clinical trials to evaluate biosimilarity, patients will receive a proposed biosimilar that is expected to be equivalent to the reference product. Some investigators and patients may have concerns about participation in clinical trials from which no therapeutic improvement is expected. There is a risk that the biosimilar may have a different efficacy or safety profile than the reference product; this is a consideration when there is a proven therapy available. Conversely, interest in clinical trials of biosimilars versus innovator drugs may be enhanced because the biosimilar is compared with an active reference product rather than a control or placebo.

Enrollment into clinical trials in the United States can be challenging, even with new innovator drugs, and among patients interested in participating in an oncology clinical trial, many may prefer to enroll in a trial of a new therapy that offers the hope of increased likelihood of a cure or control of the disease. The NCCN has cited the potential for low physician and patient interest in participating in biosimilar clinical trials as a challenge 1. However, this may not hold true for community-based clinics participating in clinical research. It should be noted that a number of biosimilars have been approved in the EU based on successful biosimilar clinical trials with adequate patient enrollment 12.

## Issues Affecting Clinical Practice

Biologics are essential for cancer treatment and supportive care; therefore, oncology practice will be affected when biosimilars enter the U.S. market as potentially less-expensive biologic competitors, requiring prescribers to make informed decisions. A recent survey of more than 400 healthcare professionals, including oncologists, indicated that most respondents had only a poor to fair understanding of biosimilars and the regulatory approval pathway (e.g., appropriate clinical study design and endpoints) for biosimilars; most respondents felt that education on biosimilars was important or very important to their clinical practice 28. Some key considerations related to the market entry and use of biosimilars in oncology are provided below.

### Interchangeability and automatic substitution

Although interchangeability will be determined by the FDA, the regulation of therapeutic interchange and automatic substitution is controlled by state pharmacy boards and state laws, which may vary between states 1,29. All state laws currently permit a physician to specify that a prescription should be dispensed as written; however, pharmacist substitution practices vary widely by state for prescriptions for which a physician has not specified this requirement. Ultimately, medical staff and pharmacy and therapeutic (P&T) committees will decide on clinical use of a given biosimilar within their institution, including policies about automatic substitution. Most biologics for the treatment of cancer are administered in the outpatient clinic or inpatient setting, where retail pharmacy-level substitution is not generally a consideration; however, the potential for automatic substitution by the pharmacist is a consideration for biologic supportive care drugs that are dispensed to patients for self-administration 1.

In a recent Internet-based survey of oncologists and other prescribers (*n* = 376) who practice in the United States conducted by the Alliance for Safe Biologic Medicines (ASBM; a multidisciplinary organization advocating for patient safety in biosimilars policy), 85% of respondents indicated that they prefer to have the authority to specify that a biologic should not be substituted without contacting the prescriber (e.g., by designating “do not substitute” or “dispense as written”) 30. More than 80% of prescribers considered this authority critical or very important, and nearly 80% considered it critical or very important that the prescribing physician be notified if a biologic is switched by the pharmacist.

### Extrapolation of indications

The draft guidance states that the FDA may extrapolate to an indication that has not been formally investigated for the biosimilar but is approved for the reference product 7. With scientific justification, a biosimilar that was clinically studied in one tumor type may also be approved for use in another tumor type without new clinical data. To support approval of an extrapolated indication, a biosimilar sponsor may need to demonstrate that the biosimilar has the same mechanism of action, target-binding characteristics, pharmacokinetics, and biodistribution in the clinically tested and the extrapolated indications, as well as address any expected differences in toxicity or effectiveness. Some concerns have been raised by biosimilar experts about the efficacy and safety of biosimilars in extrapolated indications that have not been formally evaluated in clinical studies 31. However, there are no known examples of unexpected differences in efficacy or safety in extrapolated indications for approved biosimilars in Europe compared with their reference products. The NCCN is concerned about applying biosimilar data to support off-label uses and has indicated an interest in developing recommendations regarding extrapolation in future NCCN guidelines 1. In addition, the European Group for Blood and Marrow Transplantation and the World Marrow Donor Association have expressed concern about potential extrapolation of efficacy data in the mobilization setting to biosimilar G-CSFs 32. The group recommends that biosimilar G-CSF should not be used for mobilization in normal donors outside a clinical trial given the limited safety and long-term follow-up data in this setting.

### Pharmacovigilance: safety monitoring after approval

Robust pharmacovigilance to track and accurately attribute adverse events (AEs) is important for all biologics after approval because their complexity and sensitivity to manufacturing changes make them susceptible to structural alterations that can affect safety (e.g., immunogenicity) 7,26.

Pharmacovigilance of biologics may become increasingly challenging as the number of biosimilars on the market increases. This is illustrated by an increase in the incidence of antibody-mediated pure red cell aplasia (PRCA) observed in Europe between 1998 and 2004. Ultimately, it was found to be associated with a manufacturing change that increased the immunogenicity of a marketed erythropoiesis-stimulating agent (i.e., Eprex® [epoetin alfa]) 33,34. At that time, there were only three erythropoiesis-stimulating agents on the market, yet it was challenging and time-consuming to correctly identify which erythropoiesis-stimulating agent was causing the problem. There are currently many more erythropoiesis-stimulating agents on the EU market, many of which cannot be distinguished by an international nonproprietary name (INN) alone (e.g., one epoetin alfa biosimilar is marketed under three different brand names; Table2) 12; thus, attributing PRCA to the correct product today could be even more difficult than it was 10 years ago.

Owing in part to the challenges of accurately tracking and tracing AEs of multisource biologics, the EU issued a new Pharmacovigilance Directive, which became effective in July 2012, requiring additional postmarketing safety monitoring of biologics 35,36. All biologics, including biosimilars authorized in the EU after 1 January 2011, will be identified with a black symbol in the summary of product characteristics and in the package documentation to indicate that they are subject to additional monitoring 35,36. The EU directive also requires “that all appropriate measures are taken to identify clearly any biological medicinal product prescribed, dispensed, or sold in their territory which is the subject of a suspected adverse reaction report, with due regard to the name of the medicinal product […] and the batch number” 36. Further guidance clarifies that marketing authorization holders are required to include batch number, brand name, and active substance in such reports 37.

Although the FDA requirements for pharmacovigilance have not been finalized, safety monitoring of biosimilars in clinical practice will include voluntary, spontaneous reporting of AEs and medication errors to the manufacturer or FDA by healthcare professionals and patients, as well as mandatory AE reporting by manufacturers to the FDA 26,38. It is possible that robust pharmacovigilance of biosimilars could be implemented at large institutions or those with fully integrated electronic medical and pharmacy records; however, like Risk Evaluation and Mitigation Strategies for FDA-approved oncology drugs, the postmarketing safety monitoring of biosimilars is a shared responsibility of industry in association with the FDA and other health authorities worldwide.

### Biosimilar naming

The naming of biosimilars represents another potential challenge for pharmacovigilance for drugs with multiple manufacturers. The ASBM survey indicated that more than 75% of prescribers perceived products with the same INN as structurally identical, and that nearly 70% of prescribers interpreted a shared nonproprietary name to mean that a patient could receive either product safely and expect the same results 30. Effective pharmacovigilance requires that all biologics within a product class can be distinguished from each other to facilitate accurate tracking of products and tracing of AEs to the correct product manufacturer.

Currently, there is no consensus worldwide on naming conventions for biosimilars, and the FDA has yet to define its approach. In October 2012, the World Health Organization (WHO) indicated that use of identical nonproprietary names for biosimilars may lead to inadvertent switching and that distinguishable names could be implemented by providing biosimilars with either a distinct nonproprietary name from the reference product or by assigning a unique prefix or suffix to the root nonproprietary name of the reference product 39. Current WHO policy for assigning INNs to structurally related biologics (i.e., with identical amino acid sequences) follows two different approaches, depending on whether the biologic is glycosylated 39. Nonglycosylated biologics, including biosimilars, which are considered to have posttranslational modifications that are highly similar to those of an originator product, receive the same INN. In contrast, glycosylated biologics and biosimilars, which are considered comparable but distinct from a previously approved product with the same amino acid sequence, receive the root INN of the reference product plus a Greek letter suffix to indicate different glycosylation patterns. For example, the glycosylation of epoetin zeta biosimilars differs from that of the reference product, epoetin alfa.

The WHO policy for glycosylated biologics has not been enforced consistently by the EMA, and biosimilars with different glycosylation patterns from their reference products have been authorized with the same INN 40. Examples include three biosimilar epoetin alfa products approved in 2007, as well as the first biosimilars of a monoclonal antibody (infliximab) (Table2).

### Economic considerations

Economic factors will likely play an important role in the incorporation of biosimilars into U.S. oncology practice. It has been estimated that a biosimilar will cost $100 to $200 million to develop; according to figures from DiMasi et al., an innovator biologic costs at least $800 million 41,42. The U.S. Congressional Budget Office has estimated that biosimilars will cost 20–40% less than their reference products 43, reflecting reduced testing requirements for approval. In the EU, price discounts of ∼10–35% for biosimilars have been reported, notably less than the typical 70–80% discount for generic small-molecule drugs 3,44. A recent report from the European Commission notes that biosimilars may enhance price competition for biologics, leading to reduced prices, cost savings for healthcare systems and payers, and increased patient access to biologics 45.

Oncologists, institutions, and payers will need to evaluate the potential cost savings from incorporating biosimilars into clinical practice in the context of any differences between the biosimilar and its reference product in manufacturer patient-assistance programs, out-of-pocket copayment or coinsurance costs incurred by the patient, and institutional costs associated with patient education and support. Institutions should also evaluate the need to modify their existing technology systems to facilitate accurate tracking and tracing of biologics and to ensure accurate recordkeeping and potential for prescriber notification of substitutions.

### Reliability of supply

Although generic drugs can reduce drug costs, disruptions to clinical supply have become a common occurrence in oncology practice—an ongoing concern addressed by oncology professional organizations and the FDA. The robustness of the manufacturer's supply chain is also important to consider when evaluating biosimilars, not only so that patients receive scheduled doses but also because drug shortages can lead to rationing, treatment delays, or require unplanned switching between different biologic products during a course of treatment 46–48. The majority of drug shortages are associated with inadequate quality and management in the manufacturing of the finished dosage form of the drug 49. Therefore, it will be important to consider a manufacturer's history of shortages and recalls related to quality concerns and to evaluate its capability to maintain a reliable, high-quality supply of the biosimilar. For example, more proactive manufacturers invest heavily in their inventory and the infrastructure of their supply chain to reduce the risk of drug shortages and accelerate recovery in the event of one 50.

## The NCCN Position on Biosimilars

Several U.S. professional organizations, including the NCCN, have formulated a position on biosimilars in anticipation of their approval. In March 2011, the NCCN convened a multidisciplinary Biosimilars Work Group of healthcare providers, patients, and representatives from pharmaceutical and biotechnology companies, payer organizations, and government agencies to identify challenges and provide recommendations regarding the incorporation of biosimilars into oncology practice 1. A number of challenges were identified, including the potential off-label use of biosimilars, modest cost savings, potentially low participation of physicians and patients in clinical trials, infrastructure limitations for tracking administered products in the community setting, differences in substitution practices between states and between generic small-molecule drugs and biosimilars, and the need for healthcare provider education on biosimilars. The Work Group issued a number of recommendations:

- Biosimilar clinical trials should include endpoints that are most sensitive to potential differences between the candidate biosimilar and its reference product; overall response, overall survival, and/or progression-free survival were noted as particularly helpful endpoints.
- The FDA should be consistent and transparent in how it assesses biosimilarity.
- NCCN Guidelines Panels should evaluate and provide recommendations on the clinical use of biosimilars.
- P&T committees should evaluate biosimilars considering their institutions' specific patient population (a practice not typical for generic small-molecule drugs); for healthcare professionals in practices without P&T committees, review of individual biosimilars should occur before implementing them for routine use.
- Healthcare professionals and policy makers should be educated about biosimilars.
- Guidance is needed regarding the naming of biosimilars as it relates to product tracking and safety monitoring. The Work Group issued a consensus statement noting that “The ability to track a patient's receipt of a biosimilar product during routine clinical use down to the level of a specific manufacturer and batch was seen as a critical element of assessing and ensuring the safety of these medications” 1.

Whether and how the NCCN will incorporate recommendations on biosimilars in its clinical guidelines is as yet unclear.

In April 2013, the NCCN held a summit to review advances in biosimilars since it published its recommendations 51. There was great interest in biosimilars from many different stakeholders because of their cost-saving potential, and there was recognition of the need for greater clinical experience with biosimilars, advances in technologies to characterize biologics, and education of healthcare professionals and patients on biosimilars.

## Summary

Biosimilars will be available in the United States soon and may provide alternative choices of biologic treatments for patients with cancer 1,3. There are a number of issues for oncologists to consider to make informed decisions about incorporating biosimilars into their clinical practice. Key among these issues is evaluating how substitution practices in their facility will affect patient care. Oncologists play a primary role in reporting and tracking AEs and should also consider how pharmacovigilance requirements and biosimilar naming conventions will affect their responsibilities for safety monitoring. Prescribing biosimilars may require oncologists to make changes in how they document administered products, particularly for multiple products that may be switched over the planned course of treatment. Potential cost savings from biosimilars should be evaluated in the context of differences between the biosimilar and its reference product in manufacturer patient-assistance programs, patient copayments, and institutional costs associated with education and support. When evaluating biosimilars, oncologists should also consider a manufacturer's ability to ensure a reliable, high-quality drug supply to avoid a forced and undesired switching of a patient's biologic treatment. A better understanding of these issues is important as oncologists prepare for the entry of biosimilars into clinical practice.
